# Leveraging genome characteristics to improve gene discovery for putamen subcortical brain structure

**DOI:** 10.1038/s41598-017-15705-x

**Published:** 2017-11-16

**Authors:** Chi-Hua Chen, Yunpeng Wang, Min-Tzu Lo, Andrew Schork, Chun-Chieh Fan, Dominic Holland, Karolina Kauppi, Olav B. Smeland, Srdjan Djurovic, Nilotpal Sanyal, Derrek P. Hibar, Paul M. Thompson, Wesley K. Thompson, Ole A. Andreassen, Anders M. Dale

**Affiliations:** 1 Center for Multimodal Imaging and Genetics, Department of Radiology, University of California, San Diego, La Jolla, California, 92093 USA; 2 Department of Neurosciences, University of California, San Diego, La Jolla, California, 92093 USA; 3 NORMENT, KG Jebsen Centre for Psychosis Research, Institute of Clinical Medicine, University of Oslo and Division of Mental Health and Addiction, Oslo University Hospital, Oslo, Norway; 4 Department of Cognitive Science, University of California, San Diego, La Jolla, California, 92093 USA; 5 Department of Radiation Sciences, Umea University, Umea, Sweden; 6 Department of Medical Genetics, Oslo University Hospital, Oslo, Norway; 7 NORMENT, KG Jebsen Centre for Psychosis Research, Department of Clinical Science, University of Bergen, Bergen, Norway; 8 Imaging Genetics Center, Mark and Mary Stevens Neuroimaging & Informatics Institute, Keck School of Medicine of the University of Southern California, Marina del Rey, California, 90027 USA; 9 Division of Biostatistics, Department of Family Medicine and Public Health, University of California, San Diego, La Jolla, California, 92093 USA; 10 Department of Psychiatry, University of California, SanDiego, La Jolla, California, 92093 USA

## Abstract

Discovering genetic variants associated with human brain structures is an on-going effort. The ENIGMA consortium conducted genome-wide association studies (GWAS) with standard multi-study analytical methodology and identified several significant single nucleotide polymorphisms (SNPs). Here we employ a novel analytical approach that incorporates functional genome annotations (e.g., exon or 5′UTR), total linkage disequilibrium (LD) scores and heterozygosity to construct enrichment scores for improved identification of relevant SNPs. The method provides increased power to detect associated SNPs by estimating stratum-specific false discovery rate (FDR), where strata are classified according to enrichment scores. Applying this approach to the GWAS summary statistics of putamen volume in the ENIGMA cohort, a total of 15 independent significant SNPs were identified (conditional FDR < 0.05). In contrast, 4 SNPs were found based on standard GWAS analysis (P < 5 × 10^−8^). These 11 novel loci include *GATAD2B*, *ASCC3, DSCAML*1, and *HELZ*, which are previously implicated in various neural related phenotypes. The current findings demonstrate the boost in power with the annotation-informed FDR method, and provide insight into the genetic architecture of the putamen.

## Introduction

Many aspects of the human brain, including subcortical structures, are highly heritable. Twin studies have shown genetic influences accounted for approximately 40–80% of the variance in the volume of subcortical structures^1–4^. Thus, it is unequivocal that brain structures are highly influenced by genetic factors. However, our knowledge of which genetic variants are associated with brain structural variations is currently limited. A large genome-wide association study (GWAS) by the Enhancing Neuro Imaging Genetics through Meta-Analysis (ENIGMA) consortium found the strongest effects for the putamen^3^. Four independent significant loci associated with the putamen volume were reported and all together they explained about 1.1% of phenotypic variance and 1.4–2.2% of estimated genetic variance (twin-based heritability estimated to be ~0.8^1,3^ and SNP-based heritability estimated to be ~0.5^5^), suggesting most of the genetic variance has yet to be identified.

Putamen is part of the basal ganglia, which are a group of subcortical structures involved in sensorimotor, associative, reward and mnemonic functions^6^. The dorsal part of the basal ganglia is generally associated with motor and associative functions, while the ventral part is associated with reward and motivation processes^6–8^. Dopamine is the main neurotransmitter that regulates brain activity, and the dopaminergic pathway is one of the most important anatomical substrates for reward, such as food, drugs and social interactions^9,10^, and an important pharmacological target for schizophrenia or Parkinson’s disease treatment^11^. Alterations in putamen activity or volumes have been implicated in psychiatric and substance use disorders^12–15^. However, how genes play a part in these neuroanatomical and functional characteristics remain mainly unknown. Our analysis to identify novel common genetic variants influencing the variation of putamen volume in the human population could be utilized as resources to examine genetic contribution to brain structure, function and disorders.

Large GWAS have successfully identified thousands of single nucleotide polymorphisms (SNPs) associated with hundreds of human complex traits^16,17^, thus improving our understanding of the genetic basis of many human diseases and traits. The emerging consensus from GWAS suggests that complex traits and diseases exhibit a polygenic architecture composed of many individually small effects. A polygenic architecture poses challenges for GWAS, as a massive number of statistical tests reduce power considerably for detecting small signals. As widely recognized, SNPs that exceed the GWAS significance threshold explain only a small fraction of the heritability^18,19^.

To mitigate this limitation of the standard GWAS approach, we employ a framework that concurrently uses genic annotations, heterozygosity, total linkage-disequilibrium (LD) scores, and summary statistics from GWAS^20^. We have shown that ranking SNPs according to these genome characteristics yields a larger number of loci surpassing a given threshold than ranking SNPs according to their nominal P values alone^21^. Ranking SNPs by incorporating genomic annotations and other sources of “enrichment” along with the P values obtained from existing large GWAS allows us to accelerate discovery of genetic variants associated with the phenotype of interest in a cost-efficient manner. This approach can be useful especially when phenotypes are very difficult to attain for a sufficiently large number of subjects, as is the case with brain imaging phenotypes.

The rationale behind our framework is that polymorphism variations in and around genes have been shown to harbor more genetic effects than intergenic regions^22–25^. This observation suggests that some categories of SNPs such as regulatory and coding elements of protein coding genes are more enriched for genetic effects on a phenotype than other SNPs^20,26,27^. We use our previously developed LD-weighted genic annotation method that takes into account the LD structure to select SNPs that are related to various functional categories of the genome such as exon, intron, and 3′UTR^20^. In addition to the genic annotation, we use other information in the genome to improve gene discovery, including heterozygosity (H, where H = 2 *f* (1 − *f*); *f* is allele frequency for either of the two SNP alleles) and total LD scores of individual SNPs, because variants that are of high frequency and in regions of extensive LD are more detectable in GWAS^28,29^. We integrate these various sources of enrichment information to construct a relative enrichment score (RES) for each SNP, which was used in our previous study of Covariate-Modulated Mixture Model (CM3)^21^. RES is defined as the estimated enrichment (**X** $\hat{{\boldsymbol{\beta }}}$) obtained from a logistic regression model for the thresholded GWAS summary statistics using LD-weighted genic annotation categories and total LD scores with heterozygosity weightings as explanatory variables. We then re-rank the SNPs based on their RES (instead of GWAS P values), and categorize the SNPs into several strata. For each stratum, the stratum-specific information can be used to calculate a stratified True Discovery Rate (TDR). We hypothesize that by incorporating prior enrichment information of the genome in the analysis of genotype-phenotype mapping, we can improve power to discover common genetic variants associated with the putamen volume.

## Results

### The Q-Q plot of putamen stratified by relative enrichment scores

**Figure 1:**
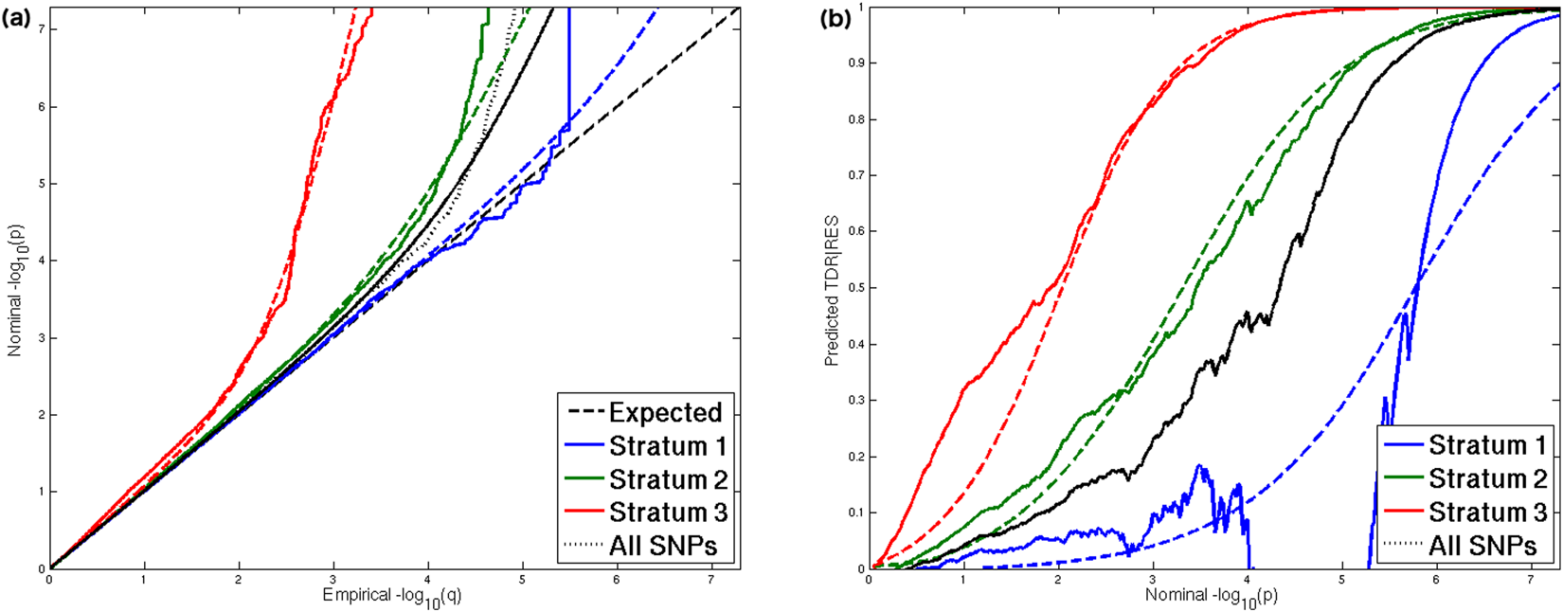
Stratified Q-Q plot of putamen volume. Stratified Q-Q and TDR plots overlaid with predicted lines show enrichment conditional on relative enrichment score (RES). (**a**) The greater degree of deflection of Q-Q curves from the expected null line is accompanied by higher level of RES strata, reflecting that SNPs in higher level of RES strata are more likely to be associated with putamen than those in lower level of RES strata. The dotted curves show predicted Q-Q curves from the mixture distribution. In each RES stratum, the Q-Q curve is fitted by using a mixture of Weibull and chi-square distributions. (**b**) TDR in each stratum is obtained from the corresponding Q-Q curve. The pattern of curves for different levels of RES is similar to the stratified Q-Q plot. It also shows that given a nominal P value, RES improves TDR estimates, indicating that stratification by RES enhances power to detect signals associated with putamen. The predicted TDR curve (dotted line) in each stratum is generated from the corresponding predicted Q-Q curve.

The stratified Q-Q plot shows different enrichment levels across RES strata, which deviate further away from the null line as RES increases (Fig. 1a). An earlier or greater departure from the null line (leftward shift) suggests a larger proportion of true associations for a given nominal P value. SNPs with higher RES (**X** $\hat{{\boldsymbol{\beta }}}$, see Methods), calculated by a logistic regression model incorporating annotation categories, total LD and heterozygosity, are more likely to be associated with putamen than those with lower RES.

### True discovery rate (TDR)

Variation in enrichment across RES strata is associated with corresponding variation in TDR for a given P value threshold (Fig. 1b). The enrichment can be directly interpreted in terms of TDR formed by estimating 1 − *P*/*Q* for each nominal P value from the stratified Q-Q plots (see Methods). This relationship is shown for putamen, the corresponding estimated TDR increases as RES stratum increases. The top RES stratum contained SNPs reaching high TDR earlier than those in other strata, indicating its greater power to identify SNPs associated with putamen.

### Predicted stratified Q-Q plot and TDR

In addition to model-free Q-Q and TDR plots generated by empirical distributions, we applied a model-based method to fit Q-Q and TDR curves in each stratum. The fitted Q-Q plot (dotted curves of Fig. 1a) is generated by using Weibull-chi-square mixture distribution (see Methods) and then the fitted TDR plot (dotted curves of Fig. 1b) is estimated by 1 − *P*/*Q* for each nominal P value as described above. Specifically, the model-based method did not perform well in Stratum 1, but had a good fitting in Stratum 2 and 3 with a larger proportion of trait-associated SNPs. In addition, curves in high TDR (i.e. low FDR) were well fit by the parametric model, which might facilitate obtaining good predicted values of TDR for detecting significant SNPs.

### Lookup table

Given nominal P values, the lookup tables were constructed by interpolated FDR conditional on RES strata (Supplementary Fig. S1b) and by interpolated FDR for all combined strata (Supplementary Fig. S1a). In the lookup table, a gradual decrease of FDR from the bottom-left to top-right corners suggests improved enrichment by stratification of RES (shown as a gradual increase of −log_10_ (*FDR*) in the figure) and smooth gradients indicate good interpolation for our FDR estimate. Given 0.05 of FDR threshold (i.e., ~1.3 for −log_10_ (*FDR*)), the corresponding nominal P value is around 10^−7^ for lower level of RES, whereas nominal P value reduces to around 10^−3^ for higher level of RES.

### P value and conditional FDR results

SNPs associated with putamen were identified by P value threshold of GWAS and FDR conditional on RES. To ensure that significant loci are independent, we removed SNPs with LD *r* ^2^ > 0.2 and retained the SNP with the lowest FDR P value in each LD block. For a GWAS threshold of P value < 5×10^−8^, a total of 4 independent SNPs located in different loci were found (Table 1). Given a threshold of conditional FDR < 0.05, we identified 15 significant independent SNPs (Table 1). Compared to the same threshold for unconditional FDR, 8 SNPs were identified. Although SNPs detected by P value are not entirely overlapping with SNPs discovered by conditional FDR, their neighboring SNPs in the same LD block (i.e., LD *r* ^2^ > 0.2 between SNPs) showed lower conditional FDR values.

**Table 1:** Genetic variants associated with putamen with conditional FDR < 0.05.

SNP	Closest gene (region)	Chr	Position	Al/A2	Frq	Beta (SE)	P value	cFDR
rs10494303	*GATAD2B* (intron)	1	153893023	G/A	0.554	24.729 (5.963)	3.37 × 10^−5^	0.0320
rs843844	*CDC73*(intergenic)	1	193271756	G/A	0.680	26.350 (6.552)	5.77 × 10^−5^	0.0486
rs17672112	*ASCC3* (intron)	6	101274689	T/C	0.803	−33.573 (7.503)	7.66 × 10^−6^	0.0254
rs610891	*AURKBPS1* (intergenic)	8	109161003	A/G	0.520	−25.636 (5.878)	1.29 × 10^−5^	0.0071
rs666845*	*DLG2* (intron)	11	83277544	C/T	0.661	−34.240 (6.084)	1.83 × 10^−8^	6.33×10^−5^
rs597583^**§**^	*DSCAML1* (intron)	11	117421799	C/G	0.805	36.894 (7.249)	3.59 × 10^−7^	0.0174
rs2181743^**§**^	*RPL7AP4* (intergenic)	14	55999725	C/T	0.148	−42.987 (7.827)	3.96 × 10^−8^	0.0081
rs8017172*	*RPL13AP3* (intergenic)	14	56199048	G/A	0.609	60.488 (5.976)	2.45 × 10^−24^	2.99 × 10^−13^
rs17253792^**§**^	*RPL13AP3* (intergenic)	14	56205030	T/C	0.936	51.776 (10.124)	3.15 × 10^−7^	0.0106
rs4788076	*SGF29* (intron)	16	28570005	C/T	0.669	26.955 (6.461)	3.02 × 10^−5^	0.0136
rs9914426	*HELZ* (intron)	17	65126641	G/C	0.508	−28.667 (5.889)	1.13 × 10^−6^	0.0272
rs12953322^**§**^	ATP7BP1 (intergenic)	18	20001349	G/A	0.513	32.246 (6.012)	8.15 × 10^−8^	0.0321
rs12457812	*DCC* (intron)	18	50444667	C/T	0.563	23.191 (6.065)	1.31 × 10^−4^	0.0448
rs11660938*	*DCC* (intron)	18	50812736	G/T	0.610	−41.504 (5.984)	4.02 × 10^−12^	3.75 × 10^−8^
rs6087771*	*BCL2L1* (intron)	20	30306724	T/C	0.675	41.038 (6.822)	1.79 × 10^−9^	5.82 × 10^−6^

A total of 15 SNPs were identified by a threshold of conditional FDR < 0.05. The SNPs with asterisk (*) are genome-wide significant, which have been reported in the original ENIGMA GWAS paper. The SNPs with section sign (^§^) are unconditional FDR significant (i.e., without incorporating annotation information). The other SNPs are additional significant SNPs identified by conditional FDR. For each intergenic SNP, the closest gene is listed. SNPs are randomly pruned using LD *r* ^2^ > 0.2 to remove correlated SNPs when estimating FDR values. All the significant loci are pruned with LD *r* ^2^ > 0.2 to report the most significant SNP in each locus. Chr: chromosome, Frq: allele frequency for A1, Beta: regression coefficient, SE: standard error of regression coefficient.

**Figure 2:**
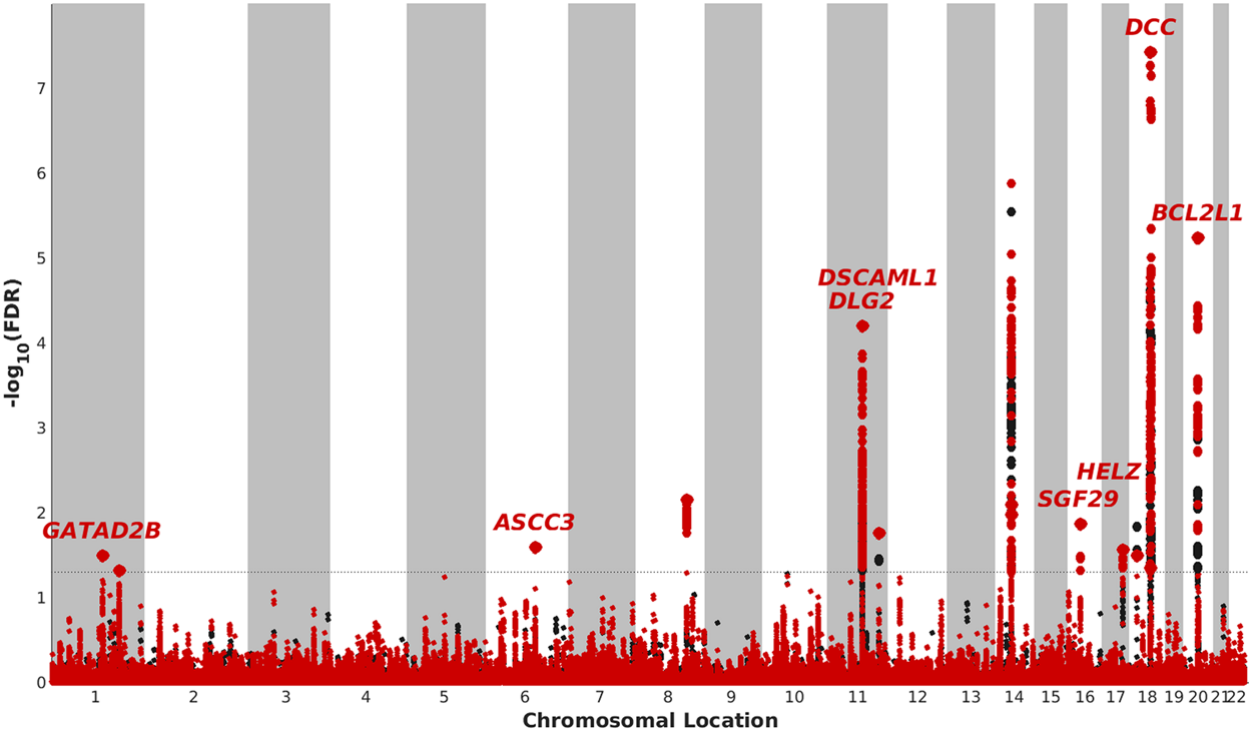
Manhattan plot for putamen volume. Manhattan plot for the putamen displays the locations of significant independent loci identified by conditional (red) and unconditional (black) −log_10_ (*FDR*) beyond the given threshold (dotted line, FDR = 0.05 and −log_10_ (*FDR*)≈1.3). The gene names are displayed for significant SNPs, except intergenic ones.

To visualize SNPs associated with putamen, we constructed a Manhattan plot showing the FDR stratified by RES. The 15 independent loci were identified with a significance threshold of conditional FDR < 0.05 (Table 1), were plotted in the Manhattan plot (Fig. 2) where gene names for those loci were also shown, except intergenic SNPs. Interestingly, several significant loci with stronger signals were distributed on chromosomes 14 and 18.

### Method comparison: fgwas

Applying fgwas to our putamen GWAS summary statistics data, only one SNP was prominent at posterior probability > 0.5, two SNPs (in the same LD block) at posterior probability > 0.4, nine SNPs (in four LD blocks) at posterior probability > 0.1. The posterior probabilities for 15 significant SNPs were shown in Supplementary Table S1 and none of SNPs with posterior probability > 0.5. The SNP rs8017172 was most significant at levels of P value, conditional FDR and posterior probability.

## Discussion

By applying a new method capitalizing on genic annotations, heterozygosity and total LD, we were able to model the cumulative probability distributions of SNPs assigned to different strata and detected 15 significant loci including 4 SNPs reported in the original GWAS paper. Using enrichment information increased the power to find more independent significant loci.

Of the 15 loci influencing putamen volume, 4 have been reported in the original GWAS paper^3^. Among 11 novel loci, we identified an intronic locus (rs597583, P = 3.59 × 10^−7^, conditional FDR = 0.0174) within *DSCAML*1 (Down syndrome cell adhesion molecule like 1), which is expressed in the brain and produces cell adhesion molecule that is involved in formation and maintenance of neural networks and neurite arborization^30,31^. The chromosomal locus of this gene on 11q23 has been suggested as a candidate for neuronal disorders^31^, because 11q23 contains a number of genes and gene families expressed in the nervous system and harbors candidate regions for several diseases with neurological features^32^. Its paralog, *DSCAM*, a conserved gene has been found to be involved in learning-related synapse formation in aplysia^33^. We also detected an intronic locus within *GATAD*2*B* (GATA zinc finger domain containing 2B), which is a protein coding gene and may play a role in synapse development and normal cognitive performance^34^. Diseases associated with *GATAD*2*B* include mental retardation and severe intellectual disability with distinct facial features^34,35^. Two other associated genes, *ASCC3* (activating signal cointegrator 1 complex subunit 3) and *HELZ* (helicase with zinc finger), encode proteins that belong to the helicase family for unwinding double-strands, which may be involved in DNA repair or RNA metabolism in multiple tissues with ubiquitous expression for *ASCC3* and predominant expression in thymus and brain for *HELZ* ^36,37^. In addition, *DLG2* identified previously^3^ and the novel locus, *ASCC3*, were reported to be suggestively associated with neurodegenerative diseases, Parkinson’s disease^38^ and multiple system atrophy^39^, respectively. The dopamine deficiency within the basal ganglia leads to Parkinsonian motor symptoms^40^ and putamen is part of the basal ganglia. In patients with Parkinson subtype of multiple system atrophy, the volume of putamen was observed to be atrophic, and MRI signals in the putamen were shown to be marginally hyperintense^41^. This evidence suggested that *DLG2* and *ASCC3* might have pleiotropic effects on putamen and neurodegenerative diseases; on the other hand, they might influence neurodegenerative diseases through alteration of putamen. Please see Table 1 for the full list of the loci discovered.

We have recently developed the FDR approach for improved gene discovery in complex genetic phenotypes^42–45^. Applying this approach to brain structure phenotypes, we increased discovery of loci jointly influencing schizophrenia and brain structure volumes^46^. The current findings suggest that re-prioritizing SNPs according to their characteristics is advantage for gene discovery in the context of FDR. It has increasingly become evident that certain genomic regions harbor more genetic effects on a given phenotype than other genomic regions^21^. The characteristic of heterozygosity for each SNP represents power to detect genetic effects in the sample population of association analysis. Heterozygosity is defined as 2 *f*(1 − *f*) and *f* is the SNP minor allele frequency, which is the genotype variance in the regression model with a higher value for common variants. It is known that allele frequency plays an important role in determining power of SNP associations^47^ (Supplementary Fig. S2), and square root of heterozygosity (H) is directly proportional to GWAS summary statistics (z values) for each SNP. We also incorporate total LD scores from the reference genomes of the same genetic ancestry (i.e. European) when calculating the relative enrichment score (RES), because if a SNP is in a large LD block, it is more likely to be linked with one or more causal variants. All of these characteristics have predictive power for genetic effects on phenotypes thus are used to construct RES for each SNP. Their enrichment features were explored and visualized in Q-Q plots (Supplementary Fig. S2). As described in Methods, RES is defined as the predicted response **X** $\hat{{\boldsymbol{\beta }}}$ from a logistic regression with these SNP characteristics as predictors, representing a composite score of estimated enrichment. All SNPs are re-ranked and stratified by their RES.

The existing methods, such as those based on stratified and conditional FDR have been shown to be superior to the traditional GWAS because they incorporate auxiliary information for stratification^47,48^. Indeed, there is often natural stratification present in the data such as stratification by allele frequency^47^ or genome annotation. Therefore, treating SNPs by strata with the incorporation of prior information, we increased the power to detect trait-associated SNPs. In comparison with fgwas^49^ which incorporates annotation information, we detected additional seven SNPs that were not identified by P value or unconditional FDR, and fgwas detected one SNP with posterior probability > 0.5 which was already significant at genome-wide P value level. It is of note that our approach is in line with the stratified FDR method^47^ (computation of FDR by strata), however, we make our FDR values continuous by computing FDR estimates on a grid and interpolating these estimates^48^. Presumably, the continuous estimates will more realistically reflect the FDR estimates for SNPs that fall in between the stratum Q-Q curves. We also build on the modeling framework initially presented in the CM3 in which the RES was formulated^21^.

There are some methodological considerations in the approach. First, although we found more significant loci than those identified by the traditional GWAS approach, all the novel loci collectively only explained a small fraction of heritability, suggesting that most of the trait-associated loci are still uncovered. Second, some parameters in our model cannot be precisely determined, for example, the total number of the strata or the percentile coverage of each stratum. This caveat is partly due to the fact that the true underlying genetic architecture of complex traits is seldom known in advance (e.g., the level of polygenicity, or annotations and allele frequencies of causal variants), and elucidating this issue is the subject of on-going research. Changes in these parameters may affect the significance status for SNPs close to the threshold, but most of the highly significant SNPs remained robust. Third, our method uses summary statistics of GWAS and hence inherits the limitations of GWAS. For example, multi-loci association analyses that take into account effects of other SNPs may give more unbiased estimates of genetic effects for each SNP. Along this line, association findings from GWAS are susceptible to the presence of population structure. Although principal components may represent broad differences across the sample, other polygenic mixed linear models including genetic relationship matrices have been proposed to be less susceptible to population structures and to increase the precision of genetic effect estimation^50,51^. Fourth, our approach is conservative formulation for FDR estimation in the enriched strata. We assume π_0_ = 1 in the model, but the top enriched stratum has lower π_0_ (i.e., lower proportion of null SNPs) so that the FDR as *P*/*Q* can be overestimated. However, other aspects in the analysis such as correlations among SNPs might over- and under-estimate FDR. We tried to minimize this issue by randomly pruning SNPs with a stringent threshold of *r* ^2^ = 0.2. We also randomly pruned the SNPs with a less stringent threshold (*r* ^2^ = 0.8). The analysis identified the same 15 loci as those from the analysis of using *r* ^2^ = 0.2. Fifth, there are other methods that use prior information. Many of these methods are Bayesian association study methods and calculate Bayes factors or posterior probability of association^49,52–54^. Some of the approaches are scalable to a very large number of functional annotations or characteristics of SNPs, and is relatively more complicated to apply in practice. Other methods propose various ways to include SNP characteristics such as multi-thresholding by varying the significant threshold at each SNP^29,55^ or defining weightings to SNPs depending on prior information via multivariate regression^56^. Our current approach is built on our prior work^20,48,57,58^, which is based on a Bayesian two-groups mixture model for Fdr control by Efron^59^. Our straightforward approach complementary to other methods can be a useful tool for gene discovery.

GWAS is an efficient tool to survey through genome-wide millions of loci for identifying any trait-associated SNPs in a hypothesis-free manner with all SNPs treated identically. As previously shown, SNPs from GWAS with sub-threshold P values account for a considerable proportion of the variance in independent samples, suggesting that these sub-threshold SNPs are enriched for genetic effects^60^. Our method is built on the GWAS approach and utilizes GWAS summary statistics in the framework of FDR as a screening tool to uncover subthreshold and high-priority candidates by incorporating genic annotations. The resulting FDR estimates may have utility as resources or databases for hypothesis generation, and could aid in more robust and meaningful candidate gene selection (e.g., testing causal genetic effects in biological experiments).

## Methods

### Participant samples

We obtained putamen GWAS results in the form of summary statistics from the ENIGMA consortium. The putamen GWAS summary statistic data consisted of 12,596 participants derived from 26 substudies with all European ancestry, which is a subset of GWAS discovery sample (N = 13,171) published in 2015^3^. Putamen GWAS was used for its better power than GWAS of other subcortical structures. All participants in substudies gave written informed consent and sites involved obtained approval from local research ethics committees or Institutional Review Boards^3^.

### Putamen structural measure

The subcortical putamen measure was obtained from structural MRI data collected, processed and examined for quality at participating sites, following a standardized protocol procedure (http://enigma.ini.usc.edu/protocols/imaging-protocols/) to harmonize the analysis across sites^3^. In addition, the measure of head size (intracranial volume, ICV) were calculated and corrected for the subcortical measures in the association analyses^3^.

### Genotyping and imputation

Samples were genotyped using commercially available platforms and assessed for genetic homogeneity using multi-dimensional scaling (MDS) analysis to exclude ancestry outliers in each substudy^3^. SNPs with low minor allele frequency (<0.01), poor genotype call rate (<95%), and deviations from Hardy–Weinberg equilibrium (P < 1 × 10^−6^) were filtered^3^. The imputation and quality control procedures were followed by the protocol (http://enigma.ini.usc.edu/protocols/genetics-protocols/) using MaCH^61^ for haplotype phasing and minimac^62^ for imputation^3^. Poorly imputed SNPs (with *r* ^2^ < 0.5) and SNPs with low minor allele count (<10) were removed. The total number of SNPs included in the analysis for each substudy ranged between 6.9–10.5 million.

### Genome-wide association analysis

The association analysis between putamen measure and each SNP (additive dosage value) was based on a multiple linear regression model controlling for age, square of age, sex, 4 MDS components, ICV, diagnosis (when applicable) and centers/scanners (for substudies with data collected from several centers/scanners)^3^. The protocols used for testing association can be found online (http://enigma.ini.usc.edu/protocols/genetics-protocols/) with mach2qtl^61^ for substudies of unrelated subjects and merlin-offline^63^ for family-based designs^3^.

### Meta-analysis of genome-wide association results from substudies

The GWAS results from each substudy were corrected for genomic inflation^3^. The meta-analysis was performed using a fixed-effect, inverse-variance model implemented in the software package METAL^64^.

SNPs for the meta-analysis were reduced into ~2.5 million SNPs based on pre-calculated LD-weighted annotation scores for individual SNPs (see the section below). The correlation structure of SNPs for calculating annotation scores was determined by an LD matrix of 2,549,449 autosomal SNPs generated from the European reference sample in the 1000 Genomes Project phase1 v3 within 1,000,000 base pairs (1 Mb)^20^.

### LD-weighted genic annotation

Each SNP analyzed in our study was annotated with LD-weighted genic annotation scores. The score was calculated based on the European reference sample provided by the November 2012 release of the Phase I 1000 Genomes Project (1KGP). Specially, each SNP in the 1KGP reference panel was initially assigned to a single mutually exclusive genic annotation category based on its genomic position (the UCSC gene database, hg19). Eight genic annotation categories were used: exon, intron, 5′ untranslated region (5′UTR), 3′UTR, 1 and 10 kilo-base pairs upstream of the gene transcription start positions, and 1 and 10 kilo-base pairs downstream of gene transcription end positions^65^. Pairwise LD scores (*r* ^2^) between SNPs were calculated. For each SNP, a continuous, non-exclusive LD-weighted category score was assigned as the LD weighted sum of the positional category scores for variants tagged in each of the eight categories mentioned above. By incorporating LD information, the annotation of individual SNPs reflects the weighted annotation in the context of underlying linkage blocks. For detailed information on SNP annotation, score construction and quality control see Schork *et al*.^20^.

### Relative enrichment score (RES)

Let p denote the P value of a particular SNP from GWAS summary statistics data. We defined *y* = 1 if p ≤ *p*_*thresh*_ (*p*_*thresh*_ = 10^−3^ in the current study) and *y* = 0 otherwise, to divide the SNPs into those that are more likely to have a non-null effect and those that are more likely to have null effects. A multiple logistic regression model was fit: logit[Pr(*y* = 1 | **X** = *x*))] = (*β*_*1*_x_*1*_ + *β*_*2*_x_*2*_ + … + *β*_*k*_ *x*_*k*_)**H**, where the *x*_*i*_, *i* = 1…*k* are the nine predictors for a SNP’s association with the phenotype. We included the genic annotation scores from the eight categories and total LD scores (TLD) weighted by heterozygosity (H = 2 *f*(1− *f*), where *f* is the SNP minor allele frequency from the 1KGP European reference panel), because they have been shown to associate with strength of association and probability of replication for many complex phenotypes^20^. The RES for the SNP is defined as the estimated value, ${\bf{X}}\hat{{\boldsymbol{\beta }}}$, from the above logistic regression model. We have used this RES approach in a previous paper^21^. Before computing the RES, SNPs were randomly pruned at LD *r* ^2^ < 0.8. Correlated SNPs do not affect *β* estimation so we prune SNPs at a liberal threshold.

GWAS summary statistics data used to calculate RES ideally should be an independent data set from the data set for gene discovery to avoid overfitting problems by fitting the same data set twice (i.e., calculating RES first and estimating conditional FDR second). However, it is often hard to obtain two or three independent GWAS data sets of a given phenotype (the third one for replication analysis). Our prior work and that of others have observed that height is extremely polygenic and its pattern of SNP associations has several typical features such as that associated signals are near genes^20,66^. Height can be used as a proxy of a generic phenotype for complex traits and its GWAS summary statistics can be used to locate polygenic loci in the genome, when multiple independent GWAS data sets of the phenotype of interest are not available. We previously adopted a similar approach using height GWAS to train the logistic regression for computing SNP enrichment scores^20,67^.

### Stratified Q-Q plots and enrichment

Q-Q plots are standard tools for assessing the degree of similarity between two cumulative distribution functions (CDFs). When the probability distribution of GWAS summary statistic P values is of interest, under the global null hypothesis, the theoretical distribution is uniform on the interval [0,1]. If nominal P values are ordered from smallest to largest, so that P(1) < P(2) < … < P(N), the corresponding empirical CDF, denoted by “Q,” is simply Q(*i*) = *i*/N, where N is the number of retained SNPs. Thus, for a given index *i*, the x-coordinate of the Q-Q curve is Q(*i*) and the y-coordinate is the nominal P value P(*i*). Instead of plotting nominal P values against empirical P values, in GWAS it is common practice to plot −log_10_ nominal P values against the −log_10_ empirical P values, Q, so as to emphasize tail probabilities of the theoretical and empirical distributions. Leftward deflections of the observed Q-Q curves from the projected null line reflect increased tail probabilities in the distribution of test statistics and consequently an over-abundance of low P values compared to that expected by chance. We qualitatively refer to this deflection as “enrichment”^20,43^.

To assess improved enrichment afforded by genic annotations, heterozygosity and total LD, we used stratified Q-Q plots based on RES. We classified SNPs with the bottom 25–30% RES as the first stratum and the SNPs with the top 1–5% RES as the last stratum. The rest of SNPs are in the second stratum. There is no overlapping set of SNPs between strata. The reason for uneven placement of stratum cut-offs at the two ends of the RES distribution was based on our previous observation that, for the distribution of effects in complex traits, a large proportion of SNPs have negligible effects and a very small proportion of SNPs have non-negligible effects. We then constructed RES stratified Q-Q plots of empirical quantiles of nominal SNP association with putamen for all SNPs, and for subsets of SNPs in each of three strata determined by their RES. Improved enrichment for trait-associated signals is present if the degree of deflection from the expected null line is dependent on the level of the RES. Specifically, the SNPs with higher RES showed a greater degree of deflection from the expected null line.

### False discovery rate

The ‘enrichment’ seen in the Q-Q plots (i.e., the leftward deflection from the null line) can be directly interpreted in terms of False Discovery Rate (FDR). To reduce the effect of correlation among SNPs in FDR estimation, SNPs were randomly pruned at LD *r* ^2^ < 0.2. For full details of FDR estimation, please see previous papers^48,57^ and Supplementary Information.

### Parametric model

The shape of the empirical distributions depicted in the Q-Q plots resembles the shape of the distribution function of a mixture of Weibull and chi-square distributions. So, for each RES stratum we modeled the Q-Q curve with a function proportional to the distribution function of a Weibull-chi-square mixture to compute stratum-specific predicted FDR. We assumed different scale parameters for the two component distributions. Further, an exploratory analysis showed that a value of 0.5 is a reliable choice for the shape parameter of the Weibull component. Keeping the shape parameter fixed at 0.5, the unknown parameters of the mixture were estimated by maximizing a cost function using unconstrained nonlinear optimization, where the cost function is proportional to the logarithm of the likelihood function of the parameters given the observed SNP distribution. The dotted line in Fig. 1a gives a graphic presentation of the predicted Q-Q curve from the mixture distribution using estimated parameters. The predicted TDR curve in each stratum is generated from the corresponding Q-Q curve and 1-FDR.

### Lookup table

We used heat maps to illustrate lookup tables to visualize variations of FDR across and within RES strata shown in Supplementary Fig. S1. Unconditional FDR is denoted as FDR obtained from the predicted Q-Q curve of all SNPs estimated by using Weibull-chi-square mixture distributions based on the 10,000-bin empirical quantile. Specifically, for each SNP the unconditional FDR value was obtained by linear interpolation from the predicted FDR values of 10,000 bins and illustrated by an unconditional FDR lookup table (Supplementary Fig. S1a) corresponding to variations of P values. Conditional FDR values^48^ were generated by bilinear interpolation from the predicted FDR values of 10,000 bins across three strata and displayed in a conditional lookup table (Supplementary Fig. S1b) reflecting RES strata against nominal P values. The values of FDR in terms of −log_10_(FDR) are illustrated by gradient colors in the lookup tables with color bars. Smooth gradients indicate good interpolation for FDR estimate of each SNP and colors varied from dark to light show enrichment improved by increasing RES.

### Manhattan plot

To illustrate the localization of the genetic markers associated with putamen conditional on RES, we constructed a ‘RES-stratified Manhattan plot’ by plotting all SNPs within an LD block in relation to their chromosomal location. All SNPs without pruning are shown as individual points but only the most significant SNP with respect to conditional FDR in each LD block is illustrated with its gene name in the plot. In each LD block, FDR values of SNPs were ranked in ascending order and SNPs that have high LD (*r* ^2^ > 0.2) with top SNPs were then removed. Thus, we retained the most significant SNP associated with putamen in each LD block. The large points and small points represent significant (FDR < 0.05) and non-significant SNPs, respectively. Two colors, red and black, denote signals from conditional and unconditional FDR, respectively. The red gene names denote the loci with FDR < 0.05.

### Method comparison using fgwas

To compare with other methods incorporating annotation information, we performed an additional analysis using fgwas^49^ (https://github.com/joepickrell/fgwas) which incorporates multiple functional annotations to inform GWAS. This method calculates the posterior probability that any given SNP is causal based on an empirical Bayes approach. We included eight annotation categories (exon, intron, 5′UTR, 3′UTR, 1 and 10 kb and 1 and 10 kb downstream) which is identical to RES in our analysis.

## Electronic supplementary material

Supplementary information
