# Supplementary material for: Leveraging genome characteristics to improve gene discovery for putamen subcortical brain structure
Source: Sci Rep. 2017 Nov 16;7:15736. doi: 10.1038/s41598-017-15705-x (PMC5691156; doi:10.1038/s41598-017-15705-x)
Supplement: Supplementary file 1 — Supplementary information [file 41598_2017_15705_MOESM1_ESM.pdf]

Leveraging genome characteristics to improve gene discovery for putamen subcortical brain structure

Chi-Hua Chen*, Yunpeng Wang, Min-Tzu Lo, Andrew Schork, Chun-Chieh Fan, Dominic Holland, Karolina Kauppi, Olav B. Smeland, Srdjan Djurovic, Nilotpal Sanyal, Derrek P. Hibar, Paul M. Thompson, Wesley K. Thompson, Ole A. Andreassen, Anders M. Dale

*Corresponding author: Chi-Hua Chen

Supplementary Information

False discovery rate

The 'enrichment' seen in the Q-Q plots (i.e., the leftward deflection from the null line) can be directly interpreted in terms of False Discovery Rate (FDR). For a given p-value cutoff, the FDR can be defined as the probability that a given SNP is null, given its observed p value[1,2].

$$\mathrm{FDR}(p) = \pi_0 F_0(p) / F(p), \qquad (1)$$

where π_0 is the proportion of null SNPs in the sample, F_0 is the null cumulative distribution function (CDF), and F is the CDF of all SNPs, both null and non-null. Under the null hypothesis, F_0 is the CDF of the uniform distribution on the unit interval [0,1], and F(p) can be estimated with empirical CDF Q, so that equation (1) reduces to

$$\mathrm{FDR}(P) = \pi_0\, P / Q, \qquad (2)$$

Replacing π_0 in equation (2) with unity gives a more conservative estimate of FDR. If π_0 is close to one, as is likely true for most GWAS, the increase is minimal. The quantity $1 - P/Q$, is hence a conservative formulation of the True Discovery Rate (TDR = 1-FDR). Given the $-\log_{10}$ of the Q-Q plots we can easily read off

$$-\log_{10}(\mathrm{FDR}(P)) \approx \log_{10}(Q) - \log_{10}(P) \qquad (3)$$

demonstrating that the FDR is directly related to the horizontal shift of the curves in the Q-Q plots from the expected line y = x, with a larger shift corresponding to a smaller FDR.

Supplementary Table S1. Posterior probabilities calculated by fgwas for genetic variants associated with putamen with conditional FDR < 0.05.

SNP	Gene Symbol	Chr	Position	Al/A2	Frq	Beta (SE)	P value	cFDR	PPA
rs10494303	GATAD2B	1	153893023	G/A	0.554	24.729 (5.963)	3.37×10^{-5}	0.0320	7.22×10^{-4}
rs843844	LINC01031-CDC73	1	193271756	G/A	0.680	26.350 (6.552)	5.77×10^{-5}	0.0486	2.97×10^{-3}
rs17672112	ASCC3	6	101274689	T/C	0.803	-33.573 (7.503)	7.66×10^{-6}	0.0254	4.69×10^{-3}
rs610891	AURKBPS1	8	109161003	A/G	0.520	-25.636 (5.878)	1.29×10^{-5}	0.0071	9.16×10^{-3}
rs666845*	DLG2	11	83277544	C/T	0.661	-34.240 (6.084)	1.83×10^{-8}	6.33×10^{-5}	2.21×10^{-7}
rs597583§	DSCAML1	11	117421799	C/G	0.805	36.894 (7.249)	3.59×10^{-7}	0.0174	0.0650
rs2181743§	ABI1P1, RPL7AP4	14	55999725	C/T	0.148	-42.987 (7.827)	3.96×10^{-8}	0.0081	1.59×10^{-16}
rs8017172*	RPL13AP3-KTN1	14	56199048	G/A	0.609	60.488 (5.976)	2.45×10^{-24}	2.99×10^{-13}	0.4642
rs17253792§	RPL13AP3-KTN1	14	56205030	T/C	0.936	51.776 (10.124)	3.15×10^{-7}	0.0106	1.50×10^{-17}
rs4788076	CCDC101	16	28570005	C/T	0.669	26.955 (6.461)	3.02×10^{-5}	0.0136	1.63×10^{-3}
rs9914426	HELZ	17	65126641	G/C	0.508	-28.667 (5.889)	1.13×10^{-6}	0.0272	0.0496
rs12953322§	ATP7BP1	18	20001349	G/A	0.513	32.246 (6.012)	8.15×10^{-8}	0.0321	0.0481
rs12457812	DCC	18	50444667	C/T	0.563	23.191 (6.065)	1.31×10^{-4}	0.0448	9.81×10^{-9}
rs11660938*	DCC	18	50812736	G/T	0.610	-41.504 (5.984)	4.02×10^{-12}	3.75×10^{-8}	0.1566
rs6087771*	BCL2L1	20	30306724	T/C	0.675	41.038 (6.822)	1.79×10^{-9}	5.82×10^{-6}	0.2072

*Genome-wide significant SNPs with P value < 5×10^{-8}

§Unconditional FDR significant SNPs with FDR < 0.05

Other significant SNPs are identified by conditional FDR < 0.05.

PPA: posterior probability of association calculated by fgwas[3].

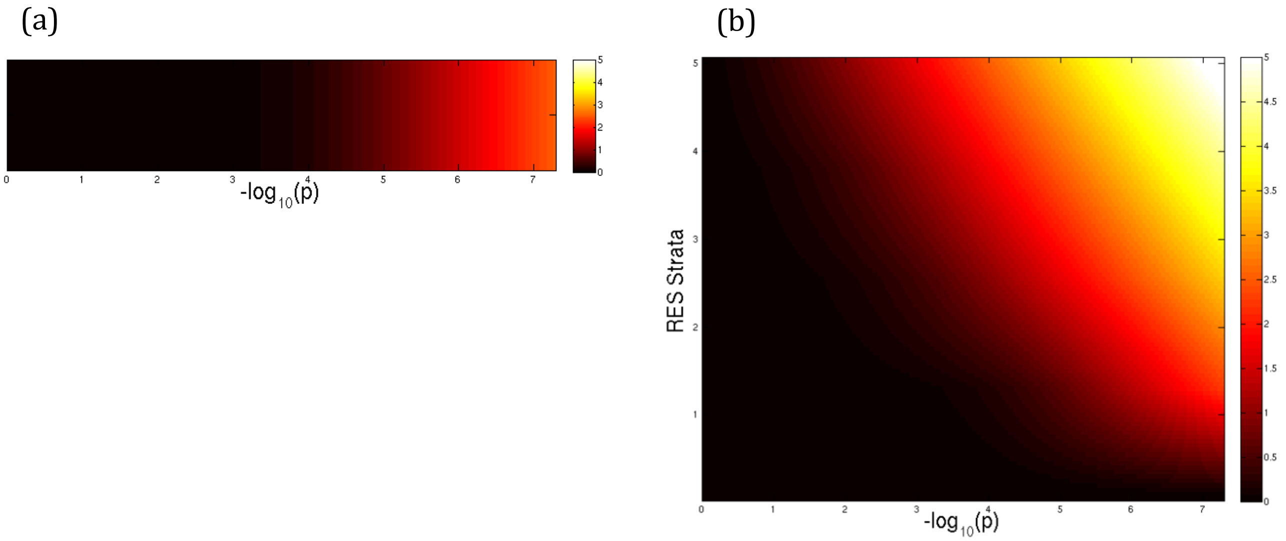

Supplementary Figure S1. FDR lookup tables.

Unconditional and conditional FDR tables in terms of negative logarithm base 10 illustrated by gradient colors in the lookup tables with color bars showing FDR values. (a) unconditional FDR for each SNP given the p-value is displayed by color scale. (b) conditional FDR for each SNP can be read from RES strata and nominal p-values.

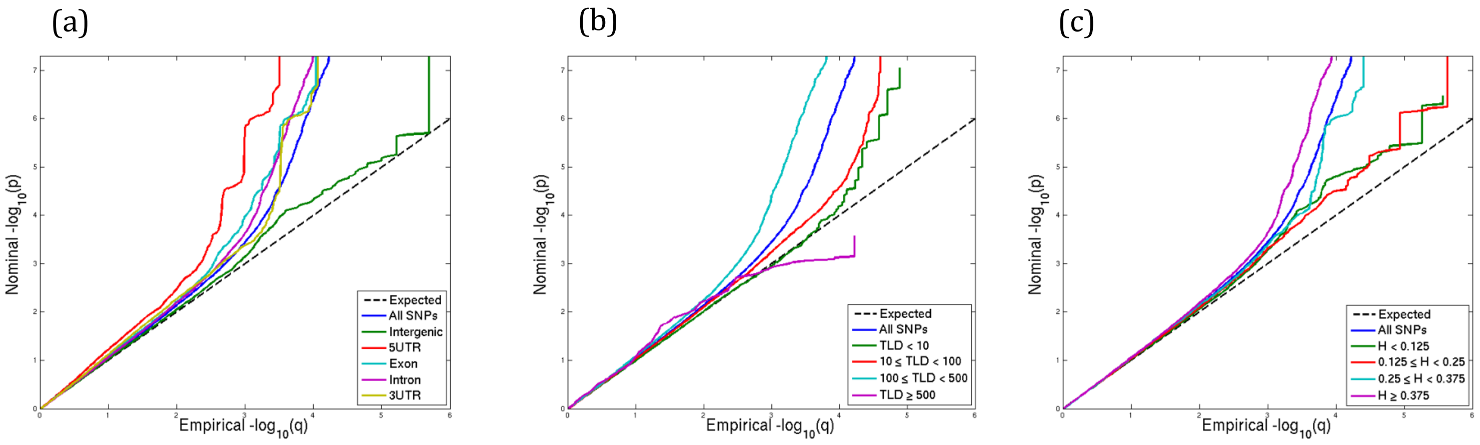

Supplementary Figure S2. Stratified Q-Q plots

Stratified Q-Q plots for putament show enrichment by (a) annotation categories, (b) total LD and (c) heterozygosity. (a) Genic annotation categories were exon, intron, 5' untranslated region (5'UTR) and 3' untranslated region (3'UTR). (b) Total LD score (TLD) for each SNP was divided into 4 strata by values of 10, 100, 500. (c) Heterozygosity (H) for each SNP was divided into 4 strata by values of 0.125, 0.25, 0.375.

References

1 Efron, B. & Tibshirani, R. Empirical bayes methods and false discovery rates for microarrays. *Genet Epidemiol* **23**, 70-86, (2002).

2 Efron, B. *Large-Scale Inference: Empirical Bayes Methods for Estimation, Testing, and Prediction* (Cambridge University Press, 2010).

3 Pickrell, J. K. Joint analysis of functional genomic data and genome-wide association studies of 18 human traits. *Am J Hum Genet* **94**, 559-573, (2014).
